# Waitlist management for inactive status kidney transplant patients: a scoping review

**DOI:** 10.1097/MS9.0000000000003137

**Published:** 2025-03-07

**Authors:** Avrey Hughes, Divyanshu Malhotra, Daniel Brennan, Lisa Seldon, Heather Carberry, Michelle Morrison, Melissa Hladek

**Affiliations:** a Johns Hopkins School of Nursing; b Johns Hopkins University; c Johns Hopkins School of Medicine; d Johns Hopkins University Medical Center; e Center on Aging and Health, Johns Hopkins University, Baltimore, Maryland

**Keywords:** inactive status, kidney transplant, waitlist management

## Abstract

Over 808 000 people live with end-stage renal disease (ESRD) in the United States. Kidney transplantation (KT) is the preferred treatment for ESRD, offering patients the best chance for long-term survival and improved quality of life compared to prolonged dependence on dialysis. There are nearly 90 000 people on the waitlist for transplant, but nearly half (47%) of patients on the KT waitlist are classified as inactive. Patients on the inactive KT waitlist have an elevated risk of post-transplant mortality and adverse outcomes. Reducing the time patients remain on the inactive KT waitlist is critical for transplant centers. Managing the waitlist for more than 250 kidney transplant programs in the US is resource and personnel intensive, and there are no best practice guidelines. Using the Johns Hopkins Nursing for Evidence-Based Practice Model as a guide, this scoping review examines the available literature on best practices for waitlist management for inactive status adult kidney transplant patients. Two themes were identified: (1) recognizing barriers to reactivation and (2) improved communication. The persistent challenges associated with inactive patients on the KT waitlist underscore the need for targeted interventions and a holistic, patient-centric approach. This review contributes to the limited literature on waitlist management for inactive KT patients and gives insights for future research, policy initiatives, and strategies to optimize the efficiency and equity of managing inactive status KT patients.

## Introduction

Over 808 000 people live with end-stage renal disease (ESRD) in the United States^[1]^. Kidney transplantation (KT) is the preferred treatment for ESRD, offering patients the best chance for long-term survival and improved quality of life compared to prolonged dependence on dialysis.^[2-4]^ The need for transplants will keep growing as 37 million people have kidney disease in the United States^[5]^. Nationally, there are over 90 000 individuals on the KT waitlist^[6]^. Longer wait times are associated with adverse outcomes such as increased waitlist mortality for KT patients^[3,7]^.

**HIGHLIGHTS**

- Over 808 000 people live with end-stage renal disease (esrd) in the united states.
- Kidney transplantation (KT) is the preferred treatment for esrd
- There are nearly 90 000 people on the waitlist for transplant, but nearly half (47%) of patients on the KT waitlist are classified as inactive.
- Patients on the inactive KT waitlist have an elevated risk of post-transplant mortality and adverse outcomes.
- Reducing the time patients remain on the inactive KT waitlist is critical for transplant centers.
- We examined the available literature on best practices for waitlist management for inactive status adult kidney transplant patients.
- This review contributes to the limited literature on waitlist management for inactive KT patients and gives insights for future research, policy initiatives, and strategies to optimize the efficiency and equity of managing inactive status KT patients.

Nearly half (47%) of patients on the KT waitlist are classified as inactive^[6]^. Inactive status indicates a patient’s ineligibility for transplant if a kidney becomes available due to medical, psychosocial, financial, or administrative factors^[7]^. In 2003, the Organ Procurement and Transplantation Network (OPTN) policy was amended to allow patients to accumulate waiting time while inactive^[8]^. Since this amendment, the number of patients designated as inactive has risen approximately five-fold^[6]^. Candidates listed as inactive tend to be older, female, identify as Black, diabetic, have poor functional status, and have a higher body mass index^[9]^. Patients who received a KT and had a prior history of inactivity had decreased rates of survival post-transplant compared to patients who were never inactive^[10]^. Patients on the inactive waitlist for KT have an elevated risk of waitlist mortality, longer wait times, and lower rates of transplantation^[9,10]^.

Inactive status is associated with a 2.2-fold increase in waitlist mortality among KT patients^[9]^. Several studies demonstrate that patients designated as inactive status experience more negative outcomes, including lower transplant rates and higher risks of death. However, it is unclear whether these outcomes are directly attributable to inactive status itself or the underlying barriers that necessitated inactivation.^[7,9-11]^. A study by Leeaphorn *et al* examined post-transplant outcomes and found that patients with a history of inactivity had lower patient and graft survival than those who were consistently active on the waitlist.^[12]^ While their findings suggest some association with inactivity, the modest survival differences (1.6%–4.7% at three years; *P* < 0.01) raise questions about the potential clinical significance and indicate that other confounding factors – such as the comorbidities or barriers that led to inactivity – may contribute to these outcomes. Further research is needed to clarify this concern.

Pre-transplant mortality is a new performance metric for evaluating transplant centers^[8]^. Effective waitlist management is a priority identified by the National Kidney Foundation^[13]^. Consequently, reducing inactive time for patients on the KT waitlist is critical for transplant centers to optimize pre-transplant metrics and improve patient outcomes. Inactive patients are at an increased risk of pre- and post-transplant mortality and adverse outcomes, underscoring the need to minimize the duration of inactive status for patients at transplant centers through evidence-based waitlist management strategies^[3,9,10]^. There is limited literature on best practices for waitlist management for inactive status adult KT patients. In response to this need, this scoping review aims to assess currently available literature for best practices in waitlist management for inactive status adult KT patients to decrease the duration of inactivity, decrease the number of inactive patients, and improve patient outcomes.

## Methods

Following the Johns Hopkins Nursing for Evidence-Based Practice Model^[14]^, four electronic databases, PubMed, Cumulative Index to Nursing and Allied Health Literature (CINAHL), Embase, and Web of Science were searched using Medical Subject Headings and associated keywords including: *waiting lists, adult,* and *kidney transplantation.* Additional keywords were: *inactive, inactivity, status 7,* and *active.* The Cochrane database was included in the search; however, it yielded only one result, which was subsequently identified as a duplicate from searches in other databases. The complete search strategies are available in the Appendix. Included articles were published between 2003 and 2024, aligning with the 2003 amendment to the OPTN policy. The author also hand-searched the reference lists of included articles and the National Kidney Foundation (NKF) for additional relevant literature.

**Figure 1.**
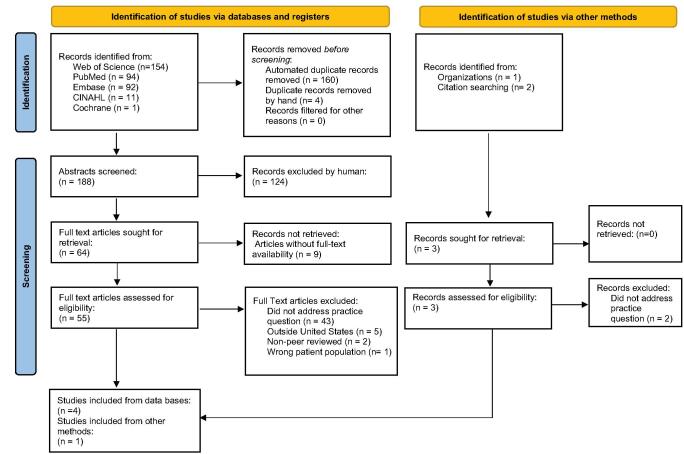
PRISMA flow diagram.

The inclusion criteria for the search included U.S.-based, English-language, peer-reviewed journal articles pertaining to the adult population over the age of 18. We included the age limit of 18 and older as pediatric patients have distinct differences in physiology, health conditions, treatment, and psychosocial factors from the adult population. Inclusion of U.S.-based articles allowed for examining context-specific interventions within the U.S. healthcare system, providing insights that can be directly applicable to the U.S. context. Articles accepted for inclusion referenced inactive status, kidney transplants, and waitlists. Articles and studies without full-text availability were excluded. A total of 354 records were identified and reduced to 189 after removing duplicates. Abstracts were screened using inclusion criteria. A total of 67 full-text articles met the inclusion criteria and were further screened for eligibility and relevance to the aim of the scoping review. After review, 5 articles met criteria for data extraction. The Preferred Reporting Items for Systematic Reviews and Meta-Analyses (PRISMA) flow diagram (Fig. 1) illustrates the search results and literature selection following established guidelines^[15]^. The characteristics of the articles were organized by research design, study participants, setting, research findings, observable measures, and evidence quality. Findings from the articles were categorized into themes using a data-driven thematic approach^[16]^. The analysis process involved assessing the consistency and relevance of the findings while identifying the themes found in the literature.

## Results

Using the Johns Hopkins Nursing Evidence-Based Practice Evidence Appraisal Tool^[14]^, five articles published between 2012 and 2021 met the inclusion criteria for appraisal. The articles included diverse levels of evidence: one classified at Level II with a B quality rating, two at Level III with an A quality rating, one at Level III with a B quality rating, and one at Level IV with a B quality rating based on the appraisal tool. Study designs included one quasi-experimental pilot study (n = 195), two retrospective cohort studies (n = 42 558 and n = 262 824), and one observational study (n = 257). Additionally, one article was a position statement from the NKF. All articles originated in the US and included two single-center studies and two studies analyzing OPTN data. All articles addressed waitlist management of inactive status patients. Additional article information is outlined in Table 1. Following review of the literature, two themes emerged: (1) recognizing barriers to reactivation and (2) improved communication.

**Table 1:** Table of evidence

Article number	Author and date	Evidence type	Sample, sample size, setting	Findings that help answer the EBP question	Observable measures	Limitations	Evidence level, quality
1	Cheng *et al*, 2018	Quasi-experimental pilot study	**Sample**: Adult KT candidates with high KAS scores	Patients managed with the TRAC system more likely to be active on the KT waitlist	Examined KT waitlist status after 18 months of TRAC system management compared to non-TRAC managed patients	Single Center Study	Level: II, Quality: B
						Study does not explore racial, cultural, or geographic factors	
			**Sample size**: 195 (active and inactive patients)				
				A higher proportion of inactive status patients managed with the TRAC system were activated compared to non-TRAC patients			
			**Setting**: Single Transplant Center				
						**Author identified**: Results may not be generalizable to other regions of the US	
2	Kataria *et al*, 2019	Observational Study	**Sample**: Adult Inactive patients on the KT waitlist	Status 7 focus group resulted in: Activation of 18% of inactive patients and subsequent transplantation	Biweekly Status 7 focus group identified individual patient barriers to activation	Single Center Study	Level: III, Quality: B
						No control group, randomization, or blinding	
			**Sample size**: 257				
			**Setting**: Single Transplant Center	Removal of 40% of inactive patients from the waitlist due to insurmountable barriers	Measured waitlist status outcomes of patients after 2 years	Results may not be generalizable to other centers	
				Time of inactivity reduced from 1344 days to 581 days (median)			
3	Kulkarni *et al*, 2019	Retrospective Cohort Study	**Sample**: Adult patients on the (OPTN) KT wait-list following the implementation of the KAS	Multistate modeling resulted in predictive insights into changes in activity status for KT patients	Determined probabilities of patient transition between active and inactive status	**Author identified**: Study design limited ability for robust casual inference	Level: III, Quality: A
						Additional factors impacting access to transplant (socioeconomic status, referral rates) were not represented	
				Communication and education on probabilities of active status increased patient/provider engagement			
			**Sample size**: 42,558				
			**Setting**: OPTN KT data base (data from 4 December 2014-8 September 2016)	Implementation of an inactive-to-active quality measure for transplant centers incentivizes improved care coordination for inactive patients			
				Resolving inactive status was higher in White patients compared to Black and Hispanic patients			
4	Lentine *et al*, 2021	Position Statement	Expert panel inlcuded nephrologists, organ procurment organization leaders, National Kidney Foundation leadership, and KT patients	Recommendations and priorities for improvement of KT patient outcomes for transplant centers inlcuded:	Expert panel reached consensus for addressing care of KT patients, improving KT waitlist access and management, and improving access to KT.	Based on expert opinions not data analysis	Level: IV, Quality: B
				Optimization of strategies to eliminate barriers for activation Focused education interventions Frequent programmatic review of inactive patients 4) Implement technology solutions to support waitlist access			
						Potential biases of the organization	
						May not be generalizable to all centers	

5	Talamantes *et al*, 2017	Retrospective cohort study	**Sample**: Adult KT transplant patients listed as inactive after the OPTN policy change in 2003	Linguistically isolated zip codes correlated with increased chances of permanent inactivity	Measured the conversion time from inactive to active status for individuals with linguistic isolation by using community-level measures	**Author identified**: Retrospective design allowed for identification of associations and did not prove causality	Level: III Quality: A
			**Sample size**: 84,783				
						Possibility of residual confounding factors	
			**Setting**: OPTN KT data base				
				“Incomplete transplant evaluation” was the predominant reason for inactivation across all groups			
				Factors including linguistic isolation, poverty, education, and Hispanic ethnicity influenced possibility of reactivation			
				Linguistic isolation impeded patient-provider communication and linked to incomplete transplant evaluations			
				Higher activation rates among White inactive KT candidates compared to Black and Hispanic candidates			

KAS, kidney allocation system; KT, kidney transplant; OPTN, Organ Procurement and Transplantation Network; Status 7 = inactive status; TRAC, transplant readiness assessment clinic.

### Recognizing barriers to reactivation

Reducing the duration of inactivity for KT patients requires identifying barriers across multiple intertwining factors including medical, psychosocial, and socioeconomic. Each of these factors includes a complex web of challenges spanning health and digital literacy, linguistic challenges, financial constraints, administrative hurdles, and social isolation, among others.^[13,17-20]^ Consequently, addressing barriers to activation consumes transplant program resources^[13,18,20]^. Ethnic and racial disparities were consistently implicated, revealing greater barriers to activation for minority KT patients compared to their white counterparts^[13,17,19,20]^. White individuals were more likely to be reactivated, while Hispanic individuals were 27% less likely and Black individuals 19% less likely to move to the active list^[13]^. Disproportionate to demographic representation, a larger percentage of white individuals are active on the waitlist compared to minority populations who have a larger representation on the inactive list^[13,19,20]^. Waitlist management strategies like the Transplant Readiness Assessment Clinic (TRAC) management system, inactive status focus groups, and utilizing inactive to active quality measures improved the identification of barriers for KT patients.^[17-19]^

### Improved communication

Consistent communication between transplant centers and inactive patients improves patient outcomes.^[13,17-20]^ Inadequate communication increases the duration of inactivity and disproportionately affects people of color and vulnerable populations.^[13,18-20]^ Inadequate communication refers to a lack of diverse and frequent touchpoints used by transplant centers to engage inactive patients and address their needs or barriers to becoming active on the waitlist. The TRAC system incorporated multiple methods of communication, including letters, multiple patient encounters, digital communication, and patient-centered education resulting in activation of 38% inactive patients versus 22–26% without TRAC management in one study of 195 people^[17]^. Implementation of inactive status focus groups by a transplant center team yielded substantial outcomes in a study of 257 people. Focus groups met biweekly for one hour and included members from all disciplines of the transplant team, including a transplant physician, social worker, financial coordinator, transplant coordinators, office assistants, and quality improvement members. Discussion at the focus groups centered on patient barriers to activation and creating medical, psychosocial, and financial action plans for each patient. Outcomes included 18% of previously inactive patients being successfully activated and transplanted, a 43% reduction in inactive time on the waitlist, and a 40% decrease in inactive patients on the waitlist due to removal for insurmountable barriers^[18]^. Shorter periods of inactivity were also attributed to communication within the transplant center team. Team communication focused on identifying barriers to activation and development of customized action plans for inactive patients.^[17-19]^

## Discussion

The high number of inactive patients on the KT waitlist continues to challenge transplant centers, resulting in increased pre- and post-transplant mortality, unfavorable surgical outcomes, and the inability of some patients to become active on the waitlist. Analysis of national trends of inactive status patients found elderly, female, and black patients were more frequently inactive than other demographics^[9]^.

Our scoping review found that recognizing the complex barriers to activation and improved patient communication decreased the time a patient is inactive. Examples of activation barriers included medical, psychosocial, financial, administrative, linguistic, digital literacy, and social isolation^[7]^. Intentional inactive patient focus groups, management systems prioritizing patient communication, personalized patient education, and frequent patient touch points positively impacted the transplant center waitlist management processes^[17,18]^. Notably, this review aligns with broader literature strongly implicating persistent ethnic and racial disparities in limited access to transplantation and disproportionate designation of inactive status. Improved communication is a central theme throughout this scoping review and broader literature addressing the issue of inactive patients on the KT waitlist. Consistent and effective communication between transplant centers and inactive patients is needed to decrease inactive time. Communication strategies, such as frequent contact with the transplant center, digital communication portals, regular patient phone calls, and targeted patient action plans decreased inactivity^[17,18]^. Implementing enhanced communication strategies may help transplant centers decrease patient inactivity and improve trust between patients and the transplant center. Transplant centers may have underutilized communication platforms, such as patient portals or reminder systems, which could be improved to provide consistent communication with inactive patients^[21]^. Improving interpersonal communication between transplant centers and patients using formal and informal contexts, such as the use of community health workers and support programs, may alleviate disparities facing vulnerable populations^[22]^.

The efficiency of kidney transplant centers is linked to their capacity and resources. As demand for kidney transplants continues to rise, managing patients on the waitlist becomes increasingly complex^[23]^. Patients who are listed as inactive status often remain inactive for an extended amount of time, highlighting the inefficiency of waitlist management practices^[11]^. Ethical considerations are important when transplant centers face limitations such as insufficient staffing, nursing resources, transplant surgeons, or organ availability. Striking a balance between providing equitable access to transplantation and managing limited resources may require difficult decisions. When demand for kidney transplants exceeds the capacity of transplant centers, the healthcare system may need to limit the waitlist. This creates a tenuous ethical situation and could disproportionately reduce access to transplantation for patients who are non-white, have lower socioeconomic status, or reside in rural areas^[24]^. Implementation of best practices for waitlist management could promote equitable access despite constraints of staffing shortages, high volume of patients, and limited resources.

The ethical considerations of the transplant process need to be explored as all parties involved navigate the delicate balance between utilitarian and deontological ethical perspectives. From a utilitarian standpoint, organs are prioritized for the “best candidate” – typically individuals with the greatest likelihood of a successful outcome. In contrast, a deontological perspective emphasizes the principles of justice and equity and questions whether the system provides equitable access to organs for all candidates, regardless of gender, socioeconomic status, racial or ethnic background. Within the ethics of the kidney transplant process, it is important to harmonize these competing ideologies – to identify the “best candidate” for each organ while also working to dismantle inequities and ensure fairness in access. Our scoping review addresses some of the deontological aspects of the transplant process when managing inactive status patients to advance justice and equity in kidney transplant care. However, more research, advocacy, and policy development are warranted.

The NKF recommends innovative strategies to optimize waitlist management for inactive KT patients through critical programmatic reviews^[13]^. Regular assessment of transplant centers’ approach to inactive KT patients could lead to the reactivation of inactive patients and removing patients who are permanently ineligible for transplant. Currently, there are no standardized national criteria for permanent removal from the KT waitlist, as these decisions are often center-specific and subject to interpretation. This review challenges transplant centers to evaluate the current methods of reviewing inactive status patients and communication between providers and inactive KT patients. Establishing targeted screenings for physical, social, psychological, or medical barriers to activation should be followed by implementing personalized action plans for patients to overcome these barriers.

The lack of best practices in managing inactive KT patients can be partly attributable to the day-to-day strain on transplant centers, including high patient volumes, insufficient staffing to manage caseloads, and inefficiencies in inactive patient review processes. Transplant centers should implement structured waitlist management practices targeting inactive KT patients. Evidence from this scoping review supports incorporating regular interdisciplinary inactive patient focus groups into transplant center workflows to identify patients who can be activated, require updated action plans, need specific barriers addressed, or should be removed from the waitlist due to insurmountable barriers. Additionally, diversifying communication methods and increasing intentional points of contact with inactive patients are evidence-based strategies. Together, these approaches may improve patient outcomes and transplant center performance metrics.

There are several strengths of this scoping review. We used a robust methodology incorporating clearly defined inclusion criteria to ensure we selected relevant and high-quality studies. Additionally, this review focuses on practical methods that can be implemented at the community and program levels. The focus on real-world, actionable solutions increases the potential impact of our findings, which are relevant for practice and policy changes focused on improving outcomes of inactive status kidney transplant patients.

We also recognize the limitations in this scoping review. A limited pool of literature was available, suggesting the need for further research on effective strategies for managing inactive status patients. Future research should focus on testing the kidney transplant waitlist management strategies identified in this review in real-world practice settings to assess the feasibility, effectiveness, and impact on patient outcomes. There is a pressing need to expand implementation science research and actively integrate evidence-based practices into the workflow of transplant centers^[25]^. The restricted scope of the search strategy may have overlooked relevant studies, potentially affecting the comprehensiveness of the findings. Additionally, the heterogeneity in the methodologies across the included studies and findings from single centers may have introduced certain biases and influenced the interpretation of the results.

## Conclusion

In summary, this review focused on best practices for waitlist management of inactive status adult KT patients to decrease the duration of inactivity and improve patient outcomes. There is a need for holistic and patient-centric approaches to managing inactive status adult KT patients. Critical programmatic review, including targeted patient reviews, can allow transplant centers to recognize and address patient barriers to activation, improve patient communication, and address equitable access to transplantation, potentially leading to decreased time on the inactive list. The findings of this review could provide guidance for future research, policy initiatives, and innovative strategies to address the management, efficiency, and equity of inactive status KT patients.

## Data Availability

None.

## Appendix

**Appendix.** Electronic database search strategy

Date of search	Database	Search strategy, terms, and keywords	# of articles found	# after filter for date of publication (2003-2024)
3/4/2024	PubMed	(((“Waiting Lists”[Mesh] OR “waiting list” [TIAB: ~ 3] OR “waiting list” [TIAB: ~ 3] OR “waitlist*” [TIAB] OR “waitlist mortality” [TIAB: ~ 3) AND (inactive [TIAB] OR inactivity [TIAB] OR active [TIAB] OR “status 7” [TIAB: ~ 3] OR “status seven” [TIAB: ~ 3])) AND (“Adult”[Mesh] OR adult* [TIAB])) AND (“Kidney Transplantation”[Mesh] OR “Kidney Transplant*” [TIAB] OR “renal transplant*” [TIAB] OR “kidney graft*” [TIAB])	99	94
3/4/2024	CINAHL	((MH “Kidney Transplantation”) OR TI (kidney N3 transplant*) OR AB (kidney N3 transplant*) OR TI (kidney N3 graft*) OR AB (kidney N3 graft*) OR TI (renal N3 transplant*) OR AB (renal N3 transplant*)) AND ((MH “Adult +”) OR TI (adult*) OR AB (adult*)) AND (TI (((wait OR waiting) N3 (list OR lists)) OR (waitlist N3 mortality) OR waitlist*) OR AB (((wait OR waiting) N3 (list OR lists)) OR (waitlist N3 mortality) OR waitlist*)) AND (TI (inactive OR inactivity OR active OR (status N3 7) OR (status N3 seven)) OR AB (inactive OR inactivity OR active OR (status N3 7) OR (status N3 seven)))	11	11
3/4/2024	Embase	(“waitlist mortality”/exp OR ((waitlist NEAR/3 mortality):ti,ab,kw) OR waitlist:ti,ab,kw OR ((waiting NEAR/3 list*):ti,ab,kw)) AND (inactive:ti,ab,kw OR inactivity:ti,ab,kw OR active:ti,ab,kw OR ((status NEAR/3 7):ti,ab,kw) OR ((status NEAR/3 seven):ti,ab,kw)) AND (“adult”/exp OR adult*:ti,ab,kw) AND (“kidney graft*”/exp OR ((kidney NEAR/3 transplant*):ti,ab,kw) OR ((kidney NEAR/3 graft*):ti,ab,kw) OR ((renal NEAR/3 transplant*):ti,ab,kw))	217	92
3/4/2024	Web of Science^a^	(((waitlist NEAR/3 mortality)) OR waitlist OR ((waiting NEAR/3 list*))) AND (inactive OR inactivity OR active OR ((status NEAR/3 7)) OR ((status NEAR/3 seven))) AND (((kidney NEAR/3 transplant*)) OR ((kidney NEAR/3 graft)) OR ((renal NEAR/3 transplant*)))	159	154
Total			486	351

Note. Cochrane database was included in the search; however, it yielded only one result, which was subsequently identified as a duplicate from searches in other databases.

^a^ In Web of Science, there is no controlled vocabulary. Therefore, the author excluded “adult” from the search strategy to mitigate the risk of overlooking relevant articles.
